# The association of mental disorders with perceived social support, and the role of marital status: results from a national cross-sectional survey

**DOI:** 10.1186/s13690-020-00476-1

**Published:** 2020-10-28

**Authors:** Janhavi Ajit Vaingankar, Edimansyah Abdin, Siow Ann Chong, Saleha Shafie, Rajeswari Sambasivam, Yun Jue Zhang, Sherilyn Chang, Boon Yiang Chua, Shazana Shahwan, Anitha Jeyagurunathan, Kian Woon Kwok, Mythily Subramaniam

**Affiliations:** 1 Research Division, Institute of Mental Health, 10, Buangkok View, Singapore, 539747 Singapore; 2 School of Social Sciences, Nanyang Technological University, 50, Nanyang Drive, Singapore, 639798 Singapore

**Keywords:** Marriage, Mediation, Multidimensional scale of perceived social support, Structural equation model, General population

## Abstract

**Background:**

This study investigated whether (i) mental disorders were associated with perceived social support and its subcomponents, (ii) current marital status was related to perceived social support, and (iii) ‘Married’ status influenced the relationship between mental state and perceived social support.

**Methods:**

Data from a cross-sectional national survey comprising 6126 respondents were used. Lifetime diagnosis for five mental disorders was assessed with a structured questionnaire. Perceived social support was measured with the Multidimensional Scale of Perceived Social Support (MSPSS) that provides Global and subscale scores for Significant Other, Family and Friends. Multiple linear regression analyses were conducted to address the research questions with MSPSS score as the dependent variable. Structural equation modeling (SEM) was performed to test mediation by marital status.

**Results:**

All mental disorders included in the study, except alcohol use disorder (AUD), were significantly and negatively associated with Global MSPSS scores. After controlling for sociodemographic factors and chronic physical illness, major depressive disorder (β = - 0.299, 95% CI: -0.484 – -0.113, *p* = 002) and having any of the five mental disorders (β = - 0.133, 95% CI: -0.254 – -0.012, *p* = 032) were negatively associated with support from Significant Other, while support from Family and Friends was lower among all disorders, except AUD. Being married was positively associated with perceived social support in people with and without mental disorders. Results of the SEM partially support mediation by mental state - perceived social support relationship by ‘Married’ status.

**Conclusion:**

Having mental disorders was associated with lower perceived social support. Being married has potential to influence this relationship.

## Background

Mental disorders affect about one-third (29.2%) of the world’s population [1], resulting in considerable global burden, disability, loss of productivity, morbidity and mortality [2, 3]. It is well known that people with mental disorders experience adverse social outcomes as they are more prone to social dysfunction, having inadequate social networks and relationship problems [4–6]. These often lead to poor received and perceived social support, which in turn is known to negatively influence symptom control, length of hospitalizations and mortality [7–9]. At the same time, it has been shown that social support can positively influence health outcomes by reducing mood-related symptoms, improving quality of life and extending life span, not just in people with mental disorders but also among those with chronic conditions such as asthma and arthritis [9, 10]. Improvements in help-seeking and treatment compliance with better perceived social support has been a particularly important development in relation to clinical outcomes in people with mental disorders [11].

Social support generally refers to social resources available to a person as result of their relationships, social circles and interactions that provide them assistance in times of need and/ or feeling of attachment. Social support is classified in terms of “structural components” such as social networks and “functional components” such as perceived social support, that is further categorized into instrumental (or tangible) and emotional (or intangible) support [12]. The sources from which individuals derive or perceive social support are diverse, and include family, friends or loved ones [13]. Support received from these different sources often varies and has distinct and overlapping pathways for health and social outcomes that could be determined by the accessibility, frequency of contact and quality of the social support. Perceived social support has been consistently linked to better health outcomes. Two mechanisms have been proposed to explain the positive effects of social ties and social support – the stress-buffering model that works when people are under stress, and the main effects model that can cause beneficial effects regardless of stress level [14]. It is believed that perceived social support acts via the former, whereby a person's perception of availability of support can enhance their help-seeking and coping mechanisms through positive appraisal of the situation and reduction in negative emotional responses. Although the exact mechanisms through which perceived social support operates are unclear [15], its effect seems to be influenced by a number of other factors, including persons’ sociodemographic background, particularly their gender and marital status [16, 17].

Marital status is the most widely investigated factor in relation to perceived social support and mental conditions given its potential as a modifiable factor [18]. Research has consistently shown that people who are married tend to have lower levels of mental disorders [19] and higher levels of perceived social support [20, 21]. compared to those who are unmarried. While one line of thought proposes that marriage in itself is a structural form of social support [16], it is also believed that marriage could be a mediator that provides social integration and feelings of belonging and purpose to individuals [17].

Singapore, a developed economy in the Asia Pacific region, has a multi-ethnic population of 5.6 million comprising 74.3% Chinese, 13.4% Malay, 9% Indian and 3.2% belonging to other ethnic groups [22]. In 2016, the prevalence of mental disorders was assessed to be 13.9% in the same adult population [23]. The study also reported higher prevalence of mood disorders among the divorced or separated population compared with those who were married. In an earlier study that assessed social distancing from people with mental disorders, 70.2% of the respondents had expressed unwillingness to have a person with mental disorder marry into their family [24]. On the other hand, another study assessing internalized stigma among people with mental disorders reported high social withdrawal and poor social relationship-related quality of life [25]. These studies indicate that people with mental disorders in Singapore are likely to have lower social resources and/or tend to be single, highlighting an unmet need to address social outcomes in mental disorders.

To the best of our knowledge, no study has been conducted among Singapore’s adult population that has investigated the relationship between mental disorders, social support and marital status. Internationally, past cross-sectional and longitudinal studies also have some limitations. In particular, most studies have assessed how poor perceived social support could result in mental conditions, or how it mediates the relationship between marital status and mental disorders. In a number of these analyses, social support was treated as an independent variable, which makes it difficult to focus on perceived social support as a dependent factor, and one that could be potentially modified and improved in people with mental disorders. The exact role played by marital status in mediating or moderating the effects of having a mental condition on perceived social support, also remains largely under explored. Moreover, social support expectations and perceptions tend to vary by cultural norms [26], and therefore, given the unique ethnic mix of the local population, it was of interest to investigate these associations in Singapore.

The current study, therefore, aimed to focus on perceived social support as a key dependent variable and investigate diverse associations between mental disorders, perceived social support and marital status. The study addressed three key research questions:

1. Are mental disorders associated with perceived social support and to which of its subcomponents?
2. Does the relationship between mental disorders and perceived social support vary in people who are currently married or single?
3. Does being married mediate the relationship between mental state and perceived social support?

## Methods

### Setting

This study was a general population-based survey in Singapore.

### Participants

The study sample included a total of 6126 Singapore residents (Citizens and Permanent Residents), aged 18 years and above, who were able to complete the survey in English, Mandarin or Malay and residing in the country during the study duration. Data was collected as part of the Singapore Mental Health Study, conducted over one and half years from August 2016. Details on the study methodology are published elsewhere [23]. Briefly, this was a cross-sectional national survey estimating prevalence of key mental disorders in Singapore. A nationally representative individual-level sample was derived from a sampling frame of all Singapore residents using disproportionate stratified sampling. For this, instead of sampling individuals accordingly to the same age and ethnic group distributions in the general population, we disproportionately sampled to obtain a sample with about 30% each of Chinese, Malays and Indians using 16 strata defined by ethnicity (Chinese, Malay, Indian, Others) and age groups (18–34, 35–49, 50–64, 65 and above). Malays and Indians (two main minority ethnic groups in Singapore) and residents aged 65 and above were over-sampled to obtain adequate numbers in these groups to improve the reliability for subgroup analyses. The oversampling proportions were then used to derive sampling weights for analysis to weight them back to the population for generalizability of the estimates.

Face to face interviews were conducted at the respondents' households by trained lay interviewers using computer assisted personal interviewing. Ethics approval was obtained from the National Healthcare Groups’ Domain Specific Review Board (Ref no: 2015/01035). Written informed consent was obtained from all respondents prior to the interviews and from a legally accepted representative for those aged below 21 years, which is the formal age of maturity in Singapore. The survey yielded a response rate of 69.5%.

### Measures

#### Diagnosis of mental disorders

The World Mental Health Composite International Diagnostic Interview (CIDI) version 3.0 was used to establish life-time Diagnostic and Statistical Manual of Mental Disorders, fourth edition (DSM-IV) diagnosis of mental disorders [27]. Given the respondent burden, only select mental disorders were included in the survey. These were major depressive disorder (MDD), dysthymia, bipolar disorder (BD), generalized anxiety disorder (GAD), obsessive-compulsive disorder (OCD) and alcohol use (alcohol abuse and dependence) disorders (AUD). Diagnosis was obtained using established algorithms with rules of hierarchy [27]. Lifetime prevalence of ‘any mental disorder’ was derived when the individuals had experienced at least one of the above conditions in their lifetime. Due to the small sample having dysthymia, it was excluded from this analysis.

#### Perceived social support

The Multidimensional Scale of Perceived Social Support (MSPSS) was used to estimate the levels of perceived social support in three domains - support from Significant Other, Family and Friends [13]. The scale comprises 12 items, with 4 items in each subscale. Respondents were asked to indicate their level of agreement to each item by using a seven-point Likert scale ranging from 1 “very strongly disagree” to 7 “very strongly agree”. Global MSPSS and domains (Significant Other, Family and Friends) scores were derived by summing the responses from the respective items, with higher scores indicating better social support. This scale has been extensively applied and validated for assessment of perceived social support, including a study among Singaporean Chinese, Malay and Indian ethnic groups [28–30]. The original English version of the scale was translated into two predominant local languages - Chinese and Malay using two independent forward translations, followed by cognitive testing. All language versions were tested in a subgroup of the local population (*n* = 15) using cognitive interviews and pre-testing to assess their acceptability. No cross-language issues were identified, and modifications were not required as the scales were well-understood and received. In this sample the internal consistency reliability for the Global MSPSS and Significant Other, Family and Friends subscales had Cronbach’s alphas of 0.91, 0.90, 0.90 and 0.93, respectively.

#### Chronic physical conditions

Respondents were asked to self-report history of chronic physical illnesses using a modified version of the CIDI chronic conditions checklist which included the following categories: (1) asthma, (2) diabetes, (3) hypertension and high blood pressure, (4) chronic pain, (5) cancer, (6) cardiovascular disorders, (7) ulcer and chronic inflamed bowel, (8) thyroid disease, (9) neurological condition, (10) chronic lung diseases, and, (11) hyperlipidemia [31]. In the current analysis, presence of any of these conditions was classified into a dichotomous variable (Yes or No).

#### Sociodemographic background

Detailed sociodemographic information was obtained from the respondents during the survey. This included age, gender (male or female), ethnicity (Chinese, Malay, Indian, or Others), marital status (never married, married, divorced/ separated or widowed), educational level (primary and below, secondary, vocational, pre-university/ junior college, diploma or university), employment status (employed, unemployed or economically inactive i.e., students, homemakers and retirees) and average monthly household income in thousand Singapore dollars (less than 2, 2–3.9, 4–5.9, 6–9.9 or 10 and over). All variables were captured for the ‘current’ state i.e. status at the time of the survey. The marital status variable was classified in three ways for the present analysis: (1) using 4 original groups i.e. never married, married, divorced/ separated or widowed, in multivariable analysis and to assess estimates in reference to married sample, (2) each group dichotomized into dummy coded variables eg, never married = 1 versus rest (all others) = 0, used to study association of specific marital status and perceived social support, and (3) marital group dummy coded as married or single (combined from never married, divorced/ separated and widowed) for assessing mediation.

### Statistical analysis

All estimates were weighted to adjust for over-sampling and non-response, and post-stratified for age and ethnicity based on the Singapore resident population of 2014. Descriptive analyses were performed to understand the socio-demographic profile of the participants and estimate Global MSPSS scores in the subgroups. Mean and standard deviation were calculated for Global MSPSS and domain scores. Multiple general linear regression models were tested to estimate (1) the association of individual mental disorders (as independent variables) with Global MSPSS and its subscale scores (as dependent variables), (2) association of marital status (as independent variables) with Global MSPSS score, and (3) association of mental disorders (as independent variables) with Global MSPSS score among the married and single, in bivariate and multivariable analysis that controlled for sociodemographic factors and diagnosis of any chronic physical condition. Statistical significance was evaluated at *p* < 0.05 using two-sided tests. These analyses were conducted using IBM SPSS version 24, Complex Samples. All estimates presented in the results section and tables are weighted estimates.

**Fig. 1:**
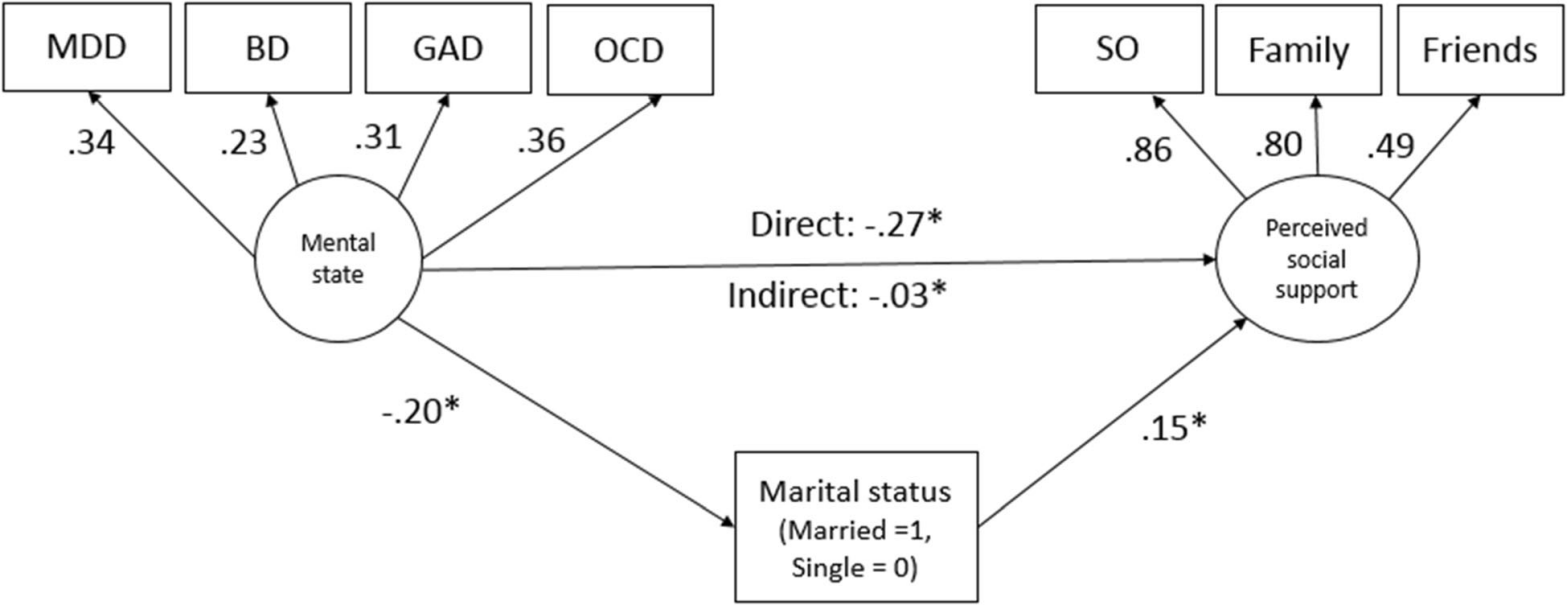
Structural equation model for mediation by marital status in the relationship between mental state and perceived social support^#^. MDD: Major depressive disorder; BD: Bipolar disorder; GAD: Generalized anxiety disorder; OCD: Obsessive compulsive disorder; SO: Significant Other. ^#^ Values in the figure are standardized regression coefficients, error terms are excluded; *p < 0.01

Structural equation modeling was performed in IBM SPSS AMOS 24.0 to test whether marital status mediated the association between mental state and social support. Marital status dummy coded as 1 = being married and 0 = single was treated as the mediating variable, latent variable mental state was the exogenous variable, while latent variable social support was the dependent variable. Latent variable for social support was derived by support from Significant Other, Family and Friends. Similarly, mental state was indicated by MDD, BD, GAD or OCD from the CIDI lifetime diagnosis. AUD was not included in this model due to lack of relationship with perceived social support. The structural equation model as illustrated (Fig. 1) was tested for indirect effects of being married (Group = 1) with a bias-corrected bootstrapping procedure based on 2000 bootstrap samples to estimate standardized regression estimates, standard errors and 95% confidence intervals. Goodness of fit indices were assessed based on following criteria: Tucker-Lewis index (TLI) and comparative fit index (CFI) close to 0.9 and Root Mean Square Error of Approximation (RMSEA) < 0.8 [32].

## Results

### Socio-demographic characteristics and perceived social support in the sample

The mean age (SD) of the participants was 45.2 (16.4) years. The sample comprised 50.4% women and 49.6% men. The sample comprised higher proportions of Chinese (75.7%), Married (59.8%) and those who were employed (72%) (Table 1). Global MSPSS score (±SD) in the overall sample was 5.59 ± 0.87. Scores (±SD) for support from Significant Other, Family and Friends in the overall sample were 5.79 ± 0.99, 5.78 ± 1.00 and 5.18 ± 1.17, respectively. Observed values for Global MSPSS scores by subgroups are included in Table 1. While Global MSPSS values varied across sociodemographic factors, these were not statistically analysed in this study.

**Table 1:** Sociodemographic background of the sample and distribution of perceived social support global scores (*n* = 6126)

	n	%	Global MSPSS score^**a**^	
			Mean	SD
**Age group**				
18–34	1707	30.4	5.73	0.80
35–49	1496	29.6	5.67	0.85
50–64	1626	26.9	5.46	0.93
65 and over	1297	13.1	5.34	0.85
**Gender**				
Men	3068	49.6	5.56	0.86
Women	3058	50.4	5.61	0.88
**Ethnicity**				
Chinese	1782	75.7	5.54	0.86
Malay	1990	12.5	5.67	0.88
Indian	1844	8.7	5.80	0.90
Others	510	3.1	5.84	0.90
**Marital status**				
Never married	1544	31.0	5.58	0.90
Married	3843	59.8	5.65	0.82
Divorced/separated	343	5.2	5.18	1.03
Widowed	396	4.1	5.23	0.89
**Education**				
Primary and below	1187	16.3	5.24	0.90
Secondary	1648	23.0	5.50	0.85
Pre-Uni./Junior College	304	6.0	5.76	0.76
Vocational	508	6.3	5.57	0.84
Diploma	1024	19.0	5.66	0.88
University	1455	29.4	5.77	0.81
**Employment**				
Employed	4055	72.0	5.62	0.83
Economically inactive	1716	22.7	5.56	0.88
Unemployed	354	5.3	5.24	1.17
**Average household income** *in Thousand Singapore dollars per month*				
Less than 2	1147	16.5	5.17	0.99
2–3.9	1331	20.0	5.49	0.87
4–5.9	1113	21.4	5.63	0.79
6–9.9	1003	21.8	5.69	0.82
10 and over	861	20.3	5.85	0.78

^a^Total score derived from the 12-item Multidimensional Scale of Perceived Social Support (MSPSS)

### Relationship between lifetime mental disorders and perceived social support

The weighted prevalence of lifetime mental disorders in the sample was 13.9%. The prevalence of MDD in the population was 6.3%, BD 1.6%, GAD 1.6%, OCD 3.6% and AUD 4.6%. In addition, 3.5% showed psychiatric comorbidity (two or more of the studied conditions). Table 2 presents results of linear regression analyses for Global MSPSS as the dependent variable. All mental disorders included in the study, except AUD, were associated with significantly lower Global MSPSS scores. Table 3 shows the association between mental disorders and independent components of perceived social support, namely support from Significant Other, Family and Friends. After controlling for sociodemographic factors and history of any chronic physical conditions, having MDD (β = - 0.299, 95% CI: -0.484 – -0.113, *p* = 0.002) or any mental disorder (β = - 0.133, 95% CI:.-0.254 – -0.012, *p* = 0.032) were significantly associated with lower support from Significant Other, while support from Family and Friends was lower among all disorders, except AUD.

**Table 2:** Relationship between lifetime mental disorders and perceived social support (Global MSPSS)

							Bivariate		Multivariable^**b**^		
	N (%)	Mean	SD	β	95% CI		*P*	β	95% CI		*P*
					Lower	Upper			Lower	Upper	
**MDD**											
Yes	346 (6.3)	5.23	1.02	−0.382	− 0.546	− 0.218	**< 0.001**	− 0.417	− 0.583	− 0.251	**< 0.001**
No	5780 (93.7)	5.61	0.85	REF				REF			
**BD**											
Yes	105 (1.6)	5.23	1.12	−0.36	− 0.665	− 0.055	**0.021**	− 0.311	− 0.611	− 0.011	**0.042**
No	6021 (98.4)	5.59	0.86	REF				REF			
**GAD**											
Yes	101 (1.6)	5.18	0.93	−0.418	−0.645	−0.192	**< 0.001**	− 0.421	− 0.658	− 0.184	**< 0.001**
No	6025 (98.4)	5.59	0.87	REF				REF			
**OCD**											
Yes	217 (3.6)	5.28	0.92	−0.319	−0.497	−0.142	**< 0.001**	− 0.368	− 0.552	− 0.184	**< 0.001**
No	5909 (96.4)	5.60	0.86	REF				REF			
**AUD**											
Yes	289 (4.6)	5.50	1.08	−0.091	−0.274	0.092	0.329	−0.042	−0.227	0.143	0.654
No	5837 (95.3)	5.60	0.86	REF				REF			
**Any mental disorder**^**a**^											
Yes	846 (13.9)	5.37	0.83	−0.249	− 0.355	− 0.143	**< 0.001**	− 0.297	− 0.408	− 0.187	**< 0.001**
No	5280 (86.1)	5.62	1.03	REF				REF			

*MDD* Major depressive disorder, *BD* Bipolar disorder, *GAD* Generalized anxiety disorder, *OCD* Obsessive compulsive disorder, *AUD* Alcohol use (abuse and dependence) disorders

^a^any of the mental disorders covered in the study (MDD, BD, GAD, OCD or AUD)

^b^Generalized linear regression models adjusted for socio-demographic characteristics (age, gender, ethnicity, marital status, education, employment, income) and having any chronic physical illness

**Table 3:** Relationship between lifetime mental disorders and domains of perceived social support

	Bivariate				Multivariable^**b**^			
	β	95% CI		*P*	β	95% CI		*P*
		Lower	Upper			Lower	Upper	
**Significant Other**								
MDD	− 0.239	− 0.419	− 0.059	**0.009**	− 0.299	− 0.484	− 0.113	**0.002**
BD	− 0.170	− 0.530	0.191	0.357	− 0.094	− 0.441	0.252	0.594
GAD	− 0.187	− 0.484	0.109	0.216	− 0.184	− 0.475	0.106	0.214
OCD	−0.148	− 0.338	0.041	0.124	−0.160	− 0.354	0.035	0.107
AUD	−0.028	−0.220	0.163	0.772	0.067	−0.130	0.265	0.505
Any mental disorder^a^	−0.121	−0.237	− 0.005	**0.040**	− 0.133	−0.254	− 0.012	**0.032**
**Family**								
MDD	−0.613	−0.809	− 0.416	**0.000**	− 0.574	−0.779	− 0.369	**0.000**
BD	−0.693	−1.131	−0.255	**0.002**	−0.396	− 0.768	−0.025	**0.037**
GAD	−0.690	−1.011	−0.368	**0.000**	−0.582	− 0.907	−0.258	**0.000**
OCD	−0.628	−0.872	− 0.385	**0.000**	− 0.498	−0.728	− 0.268	**0.000**
AUD	−0.278	−0.479	− 0.077	**0.007**	− 0.210	−0.424	0.005	0.056
Any mental disorder^a^	−0.477	−0.604	− 0.350	**0.000**	− 0.421	−0.553	− 0.290	**0.000**
**Friend**								
MDD	−0.294	−0.502	− 0.085	**0.006**	− 0.525	−0.738	− 0.313	**0.000**
BD	−0.218	−0.616	0.180	0.283	−0.420	−0.809	− 0.030	**0.035**
GAD	−0.378	−0.757	0.001	0.050	−0.492	−0.856	− 0.128	**0.008**
OCD	−0.181	−0.417	0.055	0.133	−0.442	−0.683	− 0.202	**0.000**
AUD	0.033	−0.197	0.262	0.781	0.016	−0.201	0.232	0.888
Any mental disorder^a^	−0.150	−0.287	− 0.013	**0.032**	− 0.327	−0.464	− 0.190	**0.000**

*MDD* Major depressive disorder, *BD* Bipolar disorder; GAD: Generalized anxiety disorder, *OCD* Obsessive compulsive disorder, *AUD* Alcohol use (abuse and dependence) disorders

^a^any of the mental disorders covered in the study (MDD, BDr, GAD, OCD or AUD)

^b^Generalized linear regression models adjusted for socio-demographic characteristics (age, gender, ethnicity, marital status, education, employment, income) and having any chronic physical illness

### Relationship between marital status and perceived social support

Upon adjusting for confounders, those who were never married or married showed significant negative (β = - 0.189, 95% CI: - 0.283 – -0.094, *p* < 0.001) and positive (β = 0.225, 95% CI: 0.150–0.300, *p* < 0.001) association respectively with Global MSPSS score compared to the rest. No association was observed between being widowed and perceived social support, both, in comparison with being married or the rest. Whereas being divorced/separated showed significant negative association with perceived social support versus married (β = - 0.344, 95% CI: - 0.512 –  -0.175, *p* < 0.001) and rest (β = - 0.285, 95% CI: - 0.453 –  -0.117, *p* < 0.001) (Supplementary Table 1).

### Relationship between mental disorders and perceived social support among those married and single

Results from the multiple regression models, tested to assess association between the different mental disorders and Global MSPSS score based on respondents’ current marital status (married or single) are summarized in Table 4. Regardless of the marital status of the sample, most disorders were negatively associated with perceived social support. However, among the married sample, only MDD and any mental disorder showed significant negative association with perceived social support, while all disorders except BD and AUD were negatively associated with perceived social support among the single sample.

**Table 4:** Relationship between lifetime mental disorders and perceived social support among married and single samples

	Bivariate				Multivariable^**b**^			
	β	95% CI		*P*	β	95% CI		*P*
		Lower	Upper			Lower	Upper	
**Associations among Married sample**								
MDD	−0.333	−0.583	−0.083	**0.009**	−0.505	−0.764	− 0.246	**< 0.001**
BD	−0.077	− 0.400	0.246	0.641	−0.135	− 0.491	0.221	0.457
GAD	−0.231	−0.591	0.128	0.208	−0.350	−0.707	0.008	0.055
OCD	−0.161	−0.393	0.070	0.172	−0.219	−0.467	0.029	0.083
AUD	−0.097	−0.342	0.148	0.440	−0.078	−0.332	0.176	0.548
Any mental disorder^a^	−0.160	−0.300	− 0.020	**0.025**	− 0.248	−0.397	− 0.099	**0.001**
**Associations among Single sample**								
MDD	−0.377	−0.596	− 0.159	**0.001**	− 0.437	−0.664	− 0.210	**< 0.001**
BD	−0.499	− 0.934	− 0.064	**0.025**	− 0.399	−0.817	0.018	0.061
GAD	−0.488	−0.766	− 0.21	**0.001**	− 0.464	−0.758	− 0.169	**0.002**
OCD	−0.411	−0.665	− 0.157	**0.002**	− 0.498	−0.753	− 0.243	**< 0.001**
AUD	−0.055	− 0.329	0.220	0.696	−0.013	− 0.268	0.242	0.922
Any mental disorder^a^	−0.300	−0.458	− 0.142	**< 0.001**	− 0.346	−0.507	− 0.184	**< 0.001**

^a^any of the mental disorders covered in the study (MDD, BD, GAD, OCD or AUD)

^b^Generalized linear regression models adjusted for socio-demographic characteristics (age, gender, ethnicity, education, employment, income) and having any chronic physical illness

### Mediation by marital status on the relationship between mental state and perceived social support

The structural equation model provided a good fit to the sample data with TLI = 0.895, CFI = 0.933 RMSEA = 0.062 (χ2 = 44.6, df = 18, *p* < 0.001). In the model, the path from marital status to perceived social support was significant (β = 0.15, 95% CI: 0.12–0.18, *p* < 0.01) indicating greater perceived social support among those who were Married. Significant negative association between marital status and mental state, (β = - 0.20, 95% CI: - 0.33 – -0.19, *p* < 0.01) was found. The path between mental state and perceived social support was statistically significant and negative (β = - 0.27, 95% CI: - 0.24 – -0.15, *p* < 0.01), indicating lower social support being associated with poor mental state. Significant indirect effects (β = - 0.03, 95% CI: - 0.47 – -0.21, *p* < 0.01) were seen between mental state and perceived social support through being married. Although those who had poor mental state and were married still had lower social support, the effect size of this relationship was reduced compared to the direct effect. Being married explained 11% of the mediation effect (indirect (0.03) / direct (0.27) effect size) on the relationship between mental state and perceived social support. Results indicate partial mediation by marital status.

## Discussion

The results of the study showed that common mood (MDD and BD) and anxiety (GAD and OCD) disorders were strongly related to lower perceived social support. The study also revealed that all studied mental disorders were similarly related to lower support in the domains of Family and Friends, however support from Significant Other was lower only among people with MDD. The relationship between mental disorders and perceived social support is well established [15, 32]. Perceptions that family and friends would provide effective help during times of stress have been consistently linked to good mental health and vice-versa in depression [33], posttraumatic stress disorder [34] and general psychological distress [15, 32]. Results of our study support these findings.

In the current study, MDD was the only disorder, besides overall 'any mental disorder', to be associated with lower perceived social support from Significant Other. This finding, however, should be interpreted with caution. Assessment of perceived social support from Significant Other is fraught with certain limitations, for example, whether the Significant Other is a spouse, partner or a ‘non-commitment’ relationship, perception of support from these could be influenced by social norms and culture [35]. It is also reported that gender could play a role in determining the relationship between perceived support from Significant Other and depression [36]. Hence, it is important to consider the impact of other factors on Significant Other-related social support in future studies.

The association between perceived social support and AUD is relatively under-researched and has yielded inconclusive reports. In this study, AUD did not show any relationship with perceived social support upon accounting for the effect of sociodemographic factors. Our results are similar to those observed in the National Epidemiologic Survey of Alcohol and Related Conditions conducted in USA that assessed relationships between life events, social support and alcohol consumption [37]. The study also highlighted gender-based differences whereby, under stress, women showed lower while men showed higher alcohol consumption. A study that assessed three-way relationship models of perceived social support, depression and alcohol use among adolescents found that perceived social support was negatively related to depression, which was in-turn related to alcohol use and that eventually led to decreased contact with family and friends [38]. However, these results showing the beneficial role of perceived social support in AUD have been inconsistent. While a study investigating the role of family support in American teenagers found no association with drinking behaviours [39], another found increased alcohol consumption in Chinese adults with higher perceived social support from friends [40]. The authors of the latter study attribute this observation to ingrained socio-cultural norm of strengthening friendships and social networks through drinking sessions. In our study, although not significant, support from friends and significant other was directly associated with AUD (Table 3), which needs further investigation in larger samples. Regardless of these conflicting results, it is suggested that perceived social support has a role in improving outcomes in people with AUD in terms of improving coping resources [39]. and care management [41].

Marital status emerged as an important factor while considering the association between mental disorders and perceived social support. Findings also showed that this association varies across disorders and among the married and the single, with fewer disorders showing a link with social support among the married (Table 4). Specifically, the study results highlighted the likely advantage married individuals have over the rest- comprising those never married, divorced, separated and widowed, in relation to perceived social support. These results helped confirm previous findings from similar studies. Married individuals are observed to have better mental health than unmarried, possibly due to the support perceived through marital support [42, 43]. However, it has also been reported that “being married per se is not universally beneficial” and the satisfaction and support received as part of the being in matrimony exerts “distinctive” benefits that outweigh advantages of other social networks in singles [44]. Additionally, a large study in European populations reported differences in the likelihood of mood, anxiety and personality disorders between never married and separated/divorced mothers compared to married mothers, and highlighted the relevance of investigating life and cultural contexts in interpreting these relationships [45].

In the context of Singapore’s local population – being a predominantly multi-ethnic Asian community with substantial Western influence, recent trends show that youth are not only delaying marriage, but the likelihood of singles in their mid-thirties getting married is low [46]. The author presents how gender, religion and attitudes influence how marriage is still perceived as a “revered social institution”, mainly regarded as traditional civil matrimony, as against deinstitutionalised marriages seen in Western societies, and related to “emotional intimacy”. Past research in Singapore has linked being married to lower mood disorders [23], better mental wellbeing [47] and mental health literacy [48]. Our study presents a new perspective on how the inverse association between mental health state and perceived social support could be cushioned in people who are married compared to the unmarried. However, we could not account for the effect of other social networks or investigate variations between the never married and divorced/ separated/ widowed populations due to lack of data and inadequate sample size, respectively. Future research could focus on identifying nuances in specific marital groups. Nevertheless, this study supplements the limited literature on the mediating role of marital status in the association between mental disorders and perceived social support. Results indicate that being married could be an important factor in increasing perceived social support among those with mental disorders. Studies have suggested that social ties such as marriage have a symbolic meaning attached to them that may foster a greater sense of responsibility towards healthy behaviours and improve quality of relationships and their mental health among married individuals [49]. Further research is needed to assess whether marital status directly or indirectly influences perceived social support, which has been linked to improvements in health and treatment-related outcomes such as quality of life, mood-related symptom reduction, self-management, help-seeking and treatment compliance [10, 11].

Given the likely benefits of being married with regards to perceived social support, the study also highlights a need to provide appropriate services to single people with mental disorders in order to meet their support needs. Such approaches need to be multipronged. Firstly, acknowledging the relatively poor social skills and social circles the singles with mental disorders might have, improving their social ties is of relevance. This could be done by raising awareness on social relationships, increasing civic engagement, providing opportunities and venues for developing social bonds as well as implementing measures to reduce social isolation [50, 51]. Social isolation is often also associated with unhealthy lifestyle habits, hence implementing interventions that incorporate health-related attitudes and behaviours among the unmarried may be beneficial [49]. Given the higher prevalence of mental disorders among the divorced and separated, the importance of healthy marriages should also be promoted among the married [49]. Willitts et al. [52] also suggested involving local social services and voluntary welfare organisations to provide support to people during and after separation or divorce, particularly women who could be at higher risk of developing depression. Mobilising local resources to single people with mental disorders through social and faith-based organisations could also provide social and instrumental support to individuals having lower perceived social support [52]. The study, thus, has several clinical and social service and policy implications.

An important limitation of this study is the assessment of only one aspect of social support, i.e. perceived social support. Proponents of social support research and policy have highlighted a need to include other aspects such as received social support and quality of support in the study of health [53, 54]; these parameters were not included in this survey. Secondly, the cross-sectional study design did not allow assessment of causal pathways between marriage and mental disorders and the temporal role of perceived social support on this. Thirdly, only select mental disorders were included in this study, which could have led to some misclassification of mental disorder groups. In addition, data on only current (i.e. at the time of the survey) marital status was captured, that did not allow for assessing marital transitions during persons’ lifespan which could have influenced their mental conditions and resulted in some misinterpretation. Lastly, we were unable to adopt the latest DSM-5 diagnostic criteria for this study as the updated CIDI questionnaire and algorithms were not available at the time of the survey. The study, however, has several strengths compared to earlier studies. The survey included a large representative general population sample with a comparatively high survey response rate, thus providing better precision to the data and reducing selection bias inherent in mental health surveys. In addition, a number of earlier population-based surveys focused on depression or depressive symptoms and largely used screening questionnaires such as General Health Questionnaire [55, 56], Patient Health Questionnaire [57] or Hospital Anxiety and Depression Scale [58] for assessing mental conditions. This study concurrently investigated associations for five common mental disorders that were diagnosed based on an established clinical (DSM-IV) criteria which enables direct application of the results to clinical populations. To the best of our knowledge this is the first such study conducted in an urban population that is known to have higher psychological problems [27] and where marriage-related beliefs and norms could be easily influenced by socio-economic climates. The results could also be applied to other urban settings.

## Conclusion

The current study showed that mood and anxiety disorders were associated with lower self-rated perceived social support, and that married persons were more likely to have higher perceived social support. The results partially support the notion that being married could potentially influence the negative relationship between mental disorders and perceived social support by reducing the strength of their association. Study findings should be considered while planning clinical services and social interventions for people with mental disorders. Future studies should investigate whether these effects vary by gender or other factors, and whether increased perceived social support among both, the unmarried and married could improve help-seeking and treatment compliance in people with mental disorders.

## Supplementary information

**Additional file1: Supplementary Table 1.** Relationship between marital status and perceived social support (Global MSPSS score).

## Data Availability

The data are not accessible to the public due to funding body requirements. The data may be available from the corresponding author upon reasonable request.

## Abbreviations

AUD: Alcohol Use Disorders
BD: Bipolar disorder
CFI: Comparative fit index
CI: Confidence Interval
CIDI: Composite International Diagnostic Interview
DSM-IV: Diagnostic and Statistical Manual of Mental Disorders, fourth edition
GAD: Generalized anxiety disorder
MDD: Major depressive disorder
MSPSS: Multidimensional Scale of Perceived Social Support
OCD: Obsessive-compulsive disorder
RMSEA: Root Mean Square Error of Approximation
SD: Standard deviation
SEM: Structural equation modeling
TLI: Tucker-Lewis index
