# Supplementary material for: The association of mental disorders with perceived social support, and the role of marital status: results from a national cross-sectional survey
Source: Arch Public Health. 2020 Oct 28;78:108. doi: 10.1186/s13690-020-00476-1 (PMC7592592; doi:10.1186/s13690-020-00476-1)
Supplement: Supplementary file 1 — Additional file1: Supplementary Table 1. Relationship between marital status and perceived social support (Global MSPSS score). [file 13690_2020_476_MOESM1_ESM.docx]

**Supplementary Table 1: Relationship between marital status and perceived social support (Global MSPSS score)**

|  | **Bivariate** | | | | **Multivariable#** | | | |
| --- | --- | --- | --- | --- | --- | --- | --- | --- |
|  | β | 95% CI | | P | β | 95% CI | | P |
|  |  | Lower | Upper |  |  | Lower | Upper |  |
| Never Married vs Married | -0.072 | -0.142 | -0.001 | **0.046** | -0.217 | -0.311 | -0.123 | **<0.001** |
| Divorced/separated vs Married | -0.469 | -0.635 | -0.302 | **<0.001** | -0.344 | -0.512 | -0.175 | **<0.001** |
| Widowed vs Married | -0.425 | -0.566 | -0.283 | **<0.001** | -0.109 | -0.291 | 0.073 | 0.240 |
| Married vs Rest | 0.158 | 0.093 | 0.223 | **<0.001** | 0.225 | 0.150 | 0.300 | **<0.001** |
| Never married vs Rest | -0.011 | -0.081 | 0.058 | 0.748 | -0.189 | -0.283 | -0.094 | **<0.001** |
| Divorced/separated vs Rest | -0.427 | -0.592 | -0.262 | **<0.001** | -0.285 | -0.453 | -0.117 | **0.001** |
| Widowed vs Rest | -0.376 | -0.516 | -0.236 | **<0.001** | -0.077 | -0.255 | 0.101 | 0.397 |

^#^ Generalized linear regression models adjusted for socio-demographic characteristics (age, gender, ethnicity, education, employment, income) and having any chronic physical illness
